# Derlin-1 is a target to improve radiotherapy effect of esophageal squamous cell carcinoma

**DOI:** 10.18632/oncotarget.19069

**Published:** 2017-07-07

**Authors:** Qianze Dong, Lin Fu, Yue Zhao, Yang Liu, Qingchang Li, Xueshan Qiu, Enhua Wang

**Affiliations:** ^1^ Department of Pathology, College of Basic Medical Sciences and The First Affiliated Hospital, China Medical University, Shenyang, China

**Keywords:** Derlin-1, esophageal squamous cell carcinoma, AKT, radiotherapy

## Abstract

Radiotherapy is widely used for treatment of esophageal squamous cell carcinoma (ESCC). This study aimed to explore the role of Derlin-1 on the sensitivity of ESCC to radiotherapy and its underlying mechanism. We examined the clinical significance of Derlin-1 in 125 ESCC tissues. We found that Derlin-1 protein was higher in ESCC tissues than that in normal esophageal epithelial tissues. Derlin-1 overexpression was correlated with chemoradiotherapy resistance in ESCC patients and served an independent predictor for short overall survival. siRNA knockdown and plasmid transfection were carried out in ESCC cell lines. Derlin-1 depletion inhibited cell growth while its overexpression facilitated cell growth. Derlin-1 overexpression in Eca-109 cells dramatically enhanced its resistance to radiotherapy with decreased apoptosis rate. On the contrary, Derlin-1 depletion in TE-1 cell line showed the opposite effects. In addition, radioresistance conferred by Derlin-1 was attributed to its role of activating AKT/Bcl-2 signaling pathway and reducing caspase3 cleavage. Blockage of AKT signaling attenuated the role of Derlin-1 on radioresistance. Furthermore, Derlin-1 could interact with PI3K p110α in ESCC cell lines. Taken together, Our data demonstrate that Derlin-1 overexpression predicts poor prognosis and protects ESCC from irradiation induced apoptosis through PI3K/AKT/Bcl-2 signaling pathway. Derlin-1 may serve as a novel predictor for radiosentivity and a molecular target for ESCC.

## INTRODUCTION

Esophageal squamous cell carcinoma (ESCC) is a common malignant tumor worldwide with rising incidence during the past decades [1]. The overall survival for advanced esophageal squamous cell carcinoma remains poor, with a < 30% five-year survival rate in China [2]. Chemoradiotherapy (CRT) is considered for as a standard treatment for most patients with locally advanced disease and poor response to CRT is one of the main causes of high mortality of ESCC [3–5]. Generally, esophageal squamous cell carcinomas showed an intermediate degree of sensitivity to radiotherapy. Thus, it remains important to identify novel markers that can be used to predict CRT response and prognosis of ESCC patients [6–9].

Derlin-1 was initially reported to mediate elimination of misfolded proteins from the endoplasmic reticulum (ER) [10–12]. Derlin-1 expression is elevated in breast cancer and relieves ER stress-induced apoptosis in breast cancer cells [13]. A study using tissue microarray showed that Derlin-1 was up-regulated in six types of human carcinomas, and Derlin-1-targeting antibodies suppressed colon tumor growth in isogenic mice [14]. Derlin-1 is also overexpressed in bladder and colon cancers [15, 16]. Our previous study demonstrated that Derlin-1 is overexpressed in non-small cell lung cancers and promotes invasion through regulation of EGFR activity [17]. These findings indicate that Derlin-1 serve as an oncoprotein in various human cancers. There was one report identifying microRNA-181d as a tumor suppressor in human esophageal squamous cell carcinoma by downregulating Derlin-1 [18]. However, the clinical significance of Derlin-1 in human ESCC remains explored. Its effects on therapeutic responses (including radiotherapy and chemotherapy) of cancer cells have not been elucidated.

In this study, we report Derlin-1 overexpression in ESCC tissues and cell lines associated with resistance to ionizing radiation (IR). Derlin-1 status could predict poor patient survival. We also investigated the potential molecular mechanism underlying its biological effects.

## RESULTS

### High Derlin-1 expression in esophageal squamous cell carcinoma tissues

**Figure 1:**
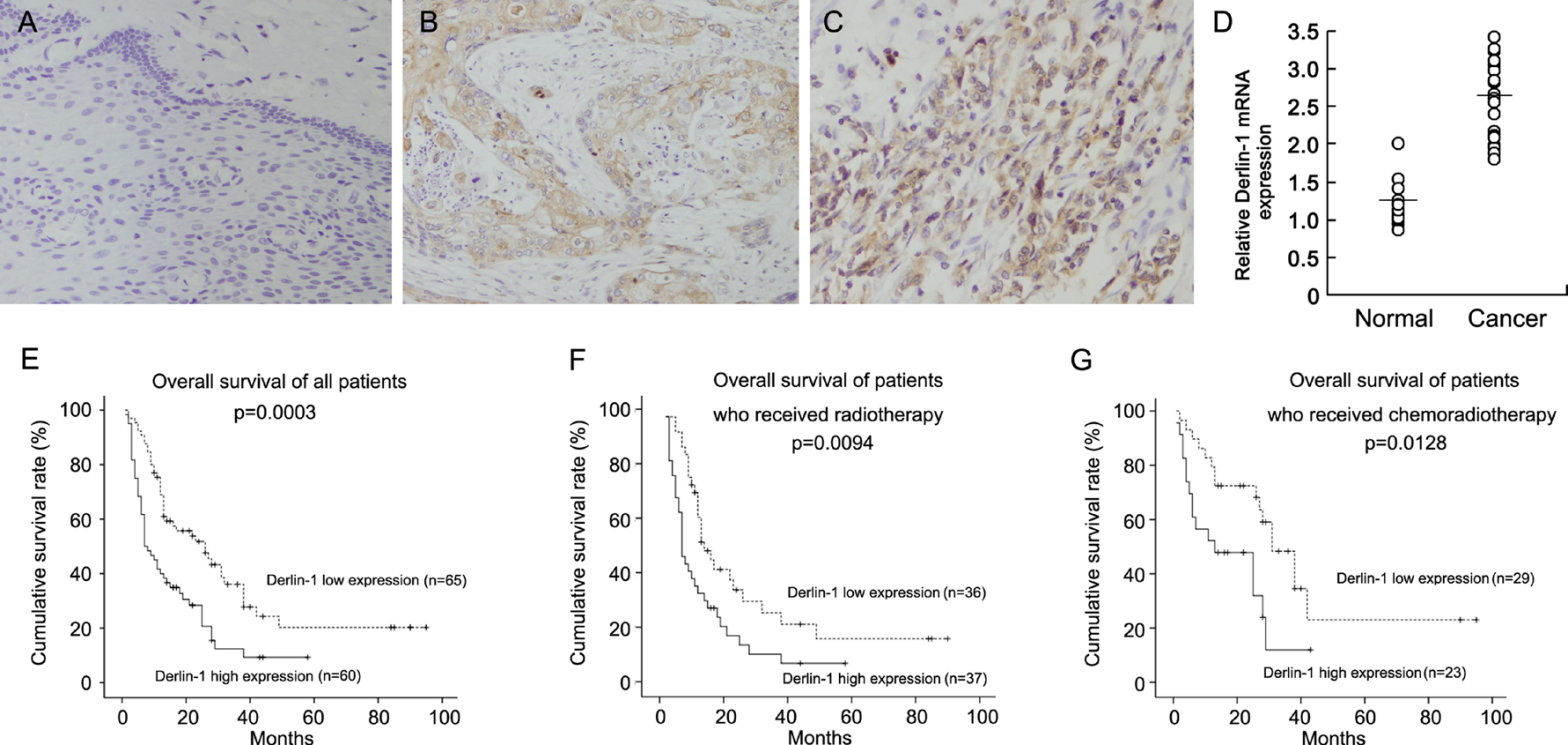
High Derlin-1 expression in esophageal squamous cell carcinoma tissues (**A**) Derlin-1 expression was negative in normal esophageal mucosa tissues. (**B**) Moderate cytoplasmic Derlin-1 expression in a case of esophageal squamous cell carcinoma tissue. (**C**) Strong cytoplasmic and nuclear Derlin-1 expression in a case of esophageal squamous cell carcinoma tissue. (**D**) Realtime RT-PCR showed that Derlin-1 mRNA was higher in cancer tissues than that in normal tissues. Short bar indicated average mRNA expression. (**E**) Overall survival of ESCC patients according to the expression of Derlin-1. (**F**) Overall survival of ESCC patients who received radiotherapy. (**G**) Overall survival of ESCC patients who received chemoradiotherapy.

We examined Derlin-1 protein in 125 cases of esophageal squamous cell carcinoma (ESCC) tissues (Figure 1A–1C). Derlin-1 expression was negative in normal esophageal mucosa tissues (Figure 1A). Cytoplasmic staining with a final score ≥ 4 was considered as Derlin-1 overexpression. Derlin-1 overexpression was observed in 60 of 125 esophageal squamous cell carcinoma tissues. We analyzed correlation of Derlin-1 with clinical factors. As shown in Table 1, no significant association was found between Derlin-1 and age, gender, differentiation, nodal metastasis and metastasis. We found that patients with a high expression of Derlin-1 tended to have a advanced TNM stage and T stage than other patients (Table 1). In addition, mRNA expression of Derlin-1 was validated in 20 cases of paired ESCC tissues with adjacent normal tissues. As shown in Figure 1D, Derlin-1 mRNA was higher in cancer tissues than that in normal tissues.

**Table 1:** Distribution of Derlin-1 status in esophageal squamous cell carcinoma according to clinicopathological characteristics

Characteristics	Number of patients	Derlin-1low expression	Derlin-1 high expression	*P*
Age				
< 60	41	20	21	0.6147
≥ 60	84	45	39	
Gender				
Female	18	11	7	0.4030
Male	107	54	53	
Differentiation				
Poor	10	4	6	0.7129
Moderate	103	55	48	
Well	12	6	6	
TNM stage				
I+II	24	18	6	0.0121
III+IV	101	47	54	
T stage				
T1–3	47	30	17	0.0399
T4	78	35	43	
Lymph node metastasis				
Negative	40	23	17	0.3985
Positive	85	42	43	
Distal metastasis				
Negative	118	63	55	0.2016
Positive	7	2	5	

Next, we analyzed the correlation between Derlin-1 with prognosis of ESCC patients. Figure 1E show the Kaplan–Meier curves of overall survival according to the expression of Derlin-1. The patients with a high Derlin-1 expression exhibited a significantly lower overall survival (*p* = 0.0003, Log-Rank test). Univariate and multivariate analyses were performed to determine the predictors of overall survival. As shown in Table 2, we found that chemotherapy, T stage and Derlin-1 overexpression as predictive markers of overall survival. Multivariate analysis showed that chemotherapy, T stage and Derlin-1 overexpression were independent risk factors for patient survival.

**Table 2:** Univariate and multivariate analysis for predictive factors in all patients with esophageal squamous cell carcinoma

	Univariate		Multivariate	
Factors	Hazard ratio (95% CI)	*p* value	Hazard ratio (95% CI)	*p* value
Differentiation	0.827 (0.487–1.404)	0.4820	1.050 (0.620–1.780)	0.8556
Chemotherapy	0.583 (0.377–0.902)	0.0153	0.517 (0.328–0.815)	0.0046
T Stage	1.673 (1.183–2.367)	0.0036	1.767 (1.184–2.637)	0.0054
Nodal status	1.200 (0.857–1.681)	0.2873	1.117 (0.768–1.626)	0.5631
Derlin-1 status	2.106 (1.385–3.201)	0.0005	1.792 (1.174–2.737)	0.0069

We divided the population into two groups by existence of adjuvant chemotherapy. Kaplan–Meier survival curve showed that Derlin-1 was associated with poor prognosis in patients radiotherapy alone (Figure 1F, *p* = 0.0094, Log-Rank test). Similar results was observed in patients who received chemoradiotherapy (Figure 1G, *p* = 0.0128, Log-Rank test). As shown in Table 3 and Table 4, high Derlin-1 status serves as independent risk factors for postoperative survival in the both groups.

**Table 3:** Univariate and multivariate analysis for predictive factors in patients with esophageal squamous cell carcinoma treated with radiotherapy

	Univariate		Multivariate	
Factors	Hazard ratio (95% CI)	*p* value	Hazard ratio (95% CI)	*p* value
Differentiation	1.468 (0.673–3.202)	0.3342	1.294 (0.498–3.367)	0.5968
T Stage	2.543 (1.521–4.251)	0.0004	2.432 (1.383–4.276)	0.0020
Nodal status	1.502 (0.918–2.457)	0.1054	1.004 (0.566–1.781)	0.9898
Derlin-1 status	1.928 (1.148–3.238)	0.0130	1.698 (1.008–2.860)	0.0467

**Table 4:** Univariate and multivariate analysis for predictive factors in patients with esophageal squamous cell carcinoma treated with chemoradiotherapy

	Univariate		Multivariate	
Factors	Hazard ratio (95% CI)	*p* value	Hazard ratio (95% CI)	*p* value
Differentiation	1.202 (0.643–2.248)	0.5639	1.097 (0.538–2.235)	0.7995
T Stage	1.386 (0.834–2.304)	0.2084	1.206 (0.711–2.046)	0.4862
Nodal status	1.223 (0.716–2.089)	0.4603	1.229 (0.719–2.100)	0.4512
Derlin-1 status	2.407 (1.169–4.956)	0.0172	2.279 (1.083–4.798)	0.0300

### Derlin-1 promotes proliferation and radioresistance of ESCC cells

**Figure 2:**
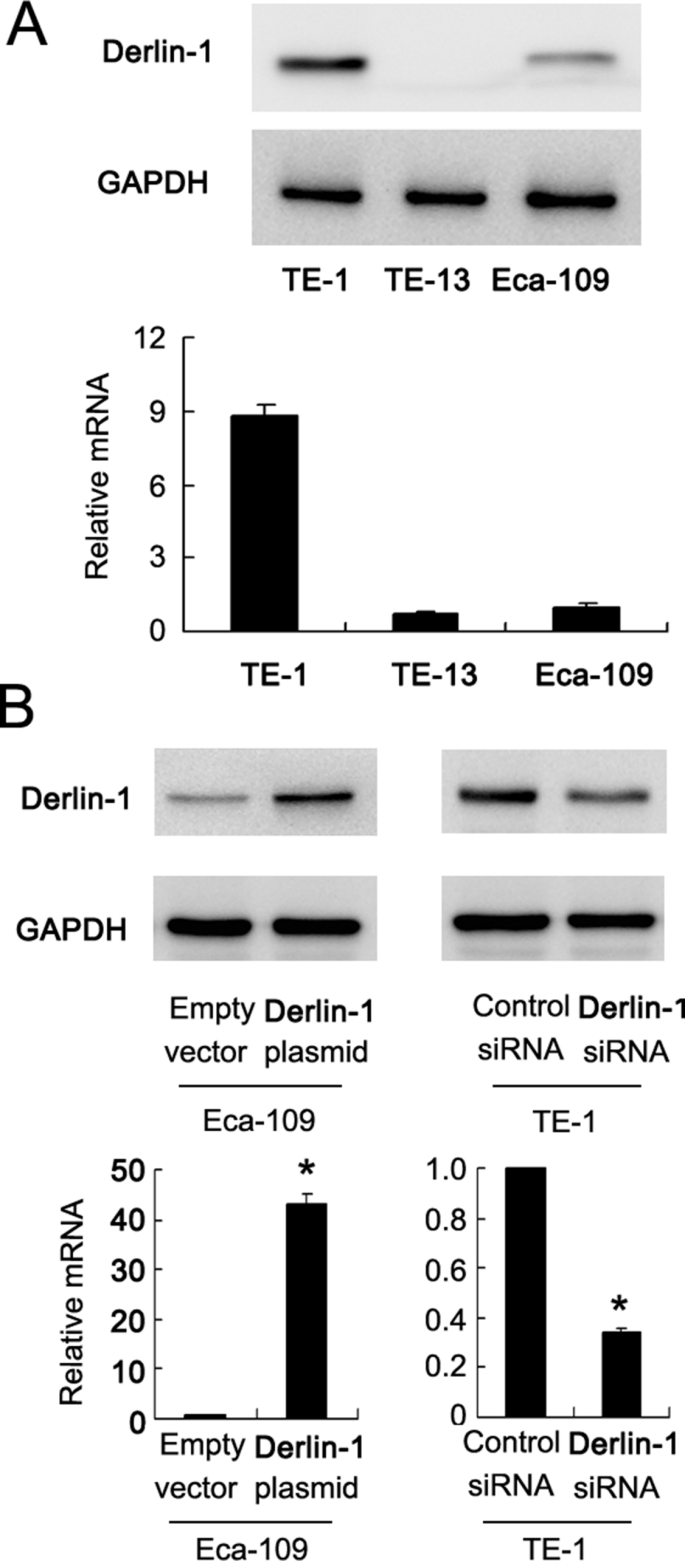
Expression of Derlin-1 in ESCC cell lines (**A**) Western blot and realtime RT-PCR showed that TE-13 and Eca-109 cell lines had low endogenous Derlin-1 expression while TE-1 cell line showed high endogenous Derlin-1 expression. (**B**) plasmid significantly upregulated Derlin-1 protein and mRNA in Eca-109 cells. siRNA significantly downregulated Derlin-1 protein and mRNA in TE-1 cells. *p < 0.05.

**Figure 3:**
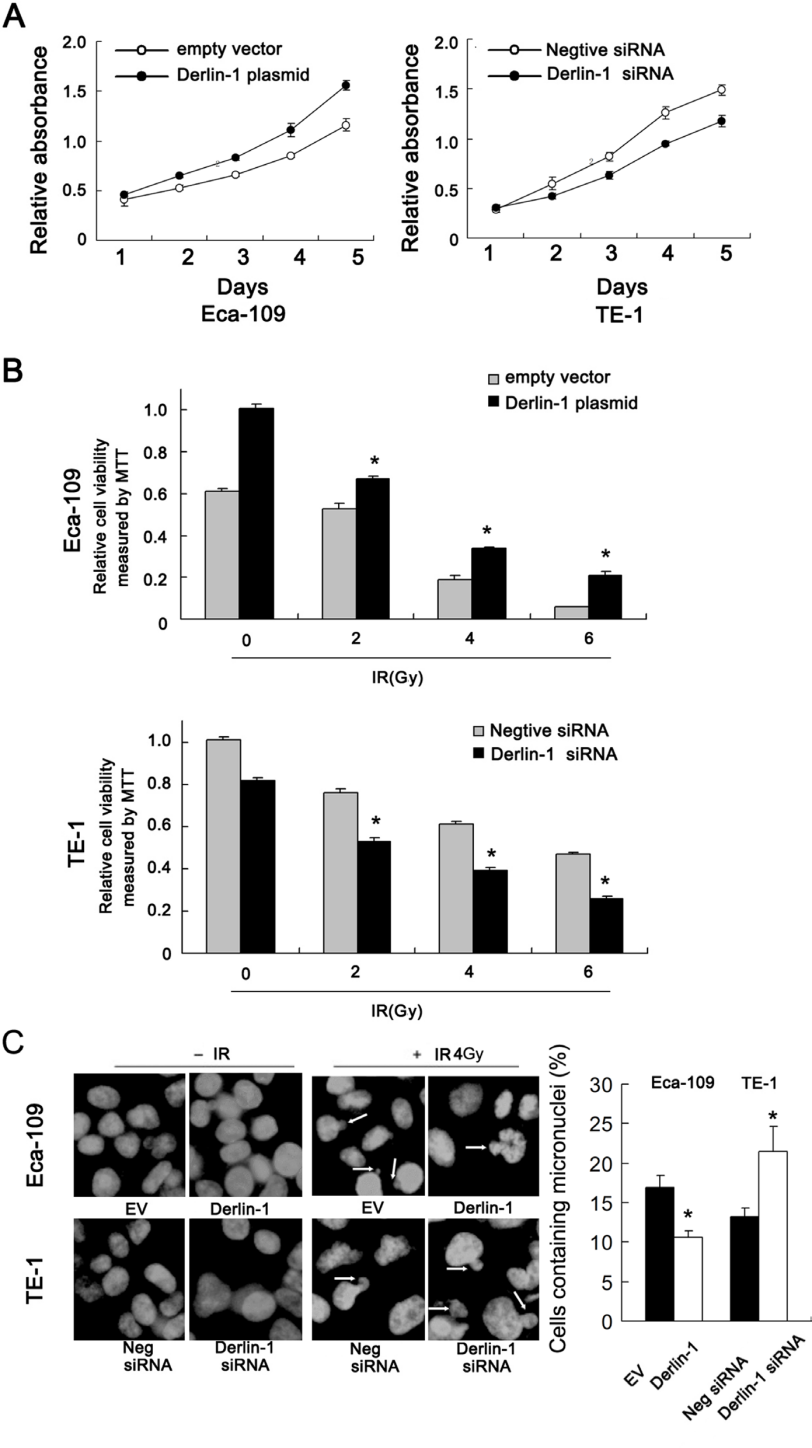
Derlin-1 promotes proliferation and radioresistance of ESCC cells (**A**) CCK-8 demonstrated that Derlin-1 transfection slightly facilitated cell proliferation while its siRNA blocked proliferation. (**B**) After 48 hours after IR treatment, the cell viability was obviously reduced in TE-1 cells treated with Derlin-1 siRNA. After Derlin-1 overexpression, the survival rate after radiation was substantially enhanced in Eca-109 cells. (**C**) Mitotic catastrophe was examined after 4 Gy radiation treatment, incidence of the micronuclear phenotype was remarkably increased in Derlin-1 depleted cells. While Derlin-1 overexpression reduced the incidence of micronuclear formation. *p < 0.05.

Protein and mRNA expression of Derlin-1 was examined by western blot in ESCC cell lines. We found that TE-13 and Eca-109 cell lines had low endogenous Derlin-1 expression while TE-1 cell line showed high endogenous Derlin-1 expression (Figure 2A). Thus, we selected Eca-109 and TE-1 to perform Derlin-1 plasmid transfection and siRNA knockdownt. As shown in Figure 2B, plasmid significantly upregulated Derlin-1 protein and mRNA in Eca-109 cells. The effect of siRNA knockdown was confirmed in TE-1 cells (Figure 2B). CCK-8 demonstrated that Derlin-1 transfection facilitated cell proliferation while its siRNA blocked proliferation (Figure 3A).

Radiotherapy is widely applied to ESCC and has a central role in the therapeutic strategy against ESCC. To explore the roles of Derlin-1 on the radiotherapy response, we examined change of cell viability, mitotic catastrophe and apoptosis following 2, 4, 6 Gy ionizing radiation (IR). As shown in Figure 3B, after 48 hours after IR treatment, the cell viability was obviously reduced in TE-1 cells treated with Derlin-1 siRNA. We also observed that after Derlin-1 overexpression in Eca-109 cells, the survival rate after radiation treatment was substantially enhanced.

### Derlin-1 reduces radiation-induced apoptosis and inhibits mitotic catastrophe

Since mitotic catastrophe and apoptosis are the 2 main forms of radiotherapy induced cell death, we explored the effects of Derlin-1 on radiation induced mitotic catastrophe. ESCC cells after 4 Gy radiation treatment. After that cells were cultured for forty-eight hours. DAPI staining showed that the incidence of the micronuclear phenotype was remarkably increased in Derlin-1 depleted cells. While Derlin-1 overexpression reduced the incidence of micronuclear formation (Figure 3C).

**Figure 4:**
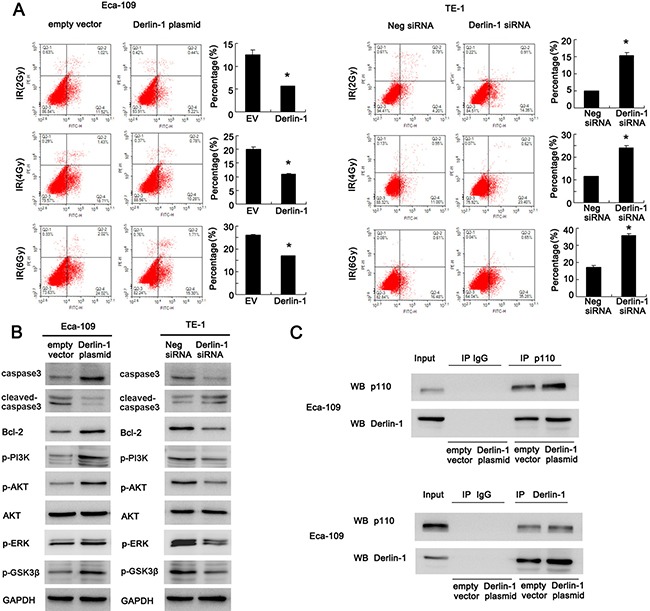
Derlin-1 inhibits apoptosis and activates AKT/Bcl-2 signaling pathway in ESCC cells (**A**) AnnexinV/PI staining showed that Derlin-1 overexpression reduced the rate of apoptosis in cells treated with 2, 4 and 6Gy radiation. Derlin-1 depletion dramatically reduced the proportion of apoptotic cells induced by radiation. (**B**) Derlin-1 depletion induced significant upregulation in cleaved caspase3 and downregulation of caspase3, Bcl-2, p-AKT, p-GSK3β, p-ERK and p-PI3K p85 in TE-1 cells treated with 4 Gy IR. In Eca-109 cells with Derlin-1 overexpression, we observed the opposite results. (**C**) Derlin-1 interacts with PI3K p110α subunit in Eca-109 cell line. *p < 0.05.

We also checked the change of apoptosis using AnnexinV/PI staining. Derlin-1 overexpression significantly reduced the rate of apoptosis in Eca-109 cells treated with 2, 4 and 6 Gy radiation. Derlin-1 depletion dramatically reduced the proportion of apoptotic TE-1 cells induced by radiation (Figure 4A).

### Derlin-1 inhibits caspase3 cleavage and activates AKT/Bcl-2 signaling pathway in ESCC cells

Consistent with previous results, Derlin-1 depletion induced significant upregulation in cleaved caspase3 in TE-1 cells treated with 4 Gy IR. Derlin-1 overexpression in Eca-109 cells reduced the level of cleaved caspase3 and upregulated total caspase3. Anti-apoptosis protein Bcl-2 and its upstream AKT signaling pathway play important roles in the development of radioresistance. Western blot revealed that Derlin-1 overexpression upregulated Bcl-2 and AKT phosphorylation (Figure 4B). In addition, Derlin-1 overexpression also upregulated p-PI3K p85, p-GSK3β and p -ERK. While Derlin-1 siRNA showed the opposite effects. Next we explored the potential molecular mechanism of Derlin-1 induced AKT activation. Using co-immunoprecipitation, we found that Derlin-1 was able to interact with PI3K p110α subunit in Eca-109 cell line. Upregulation of Derlin-1 strengthened such interaction (Figure 4C). These results indicated that Derlin-1 reduced IR induced apoptosis through PI3K/AKT/Bcl-2 signaling.

### Inhibition of AKT pathway abolishes the role of Derlin-1 on IR induced apoptosis

**Figure 5:**
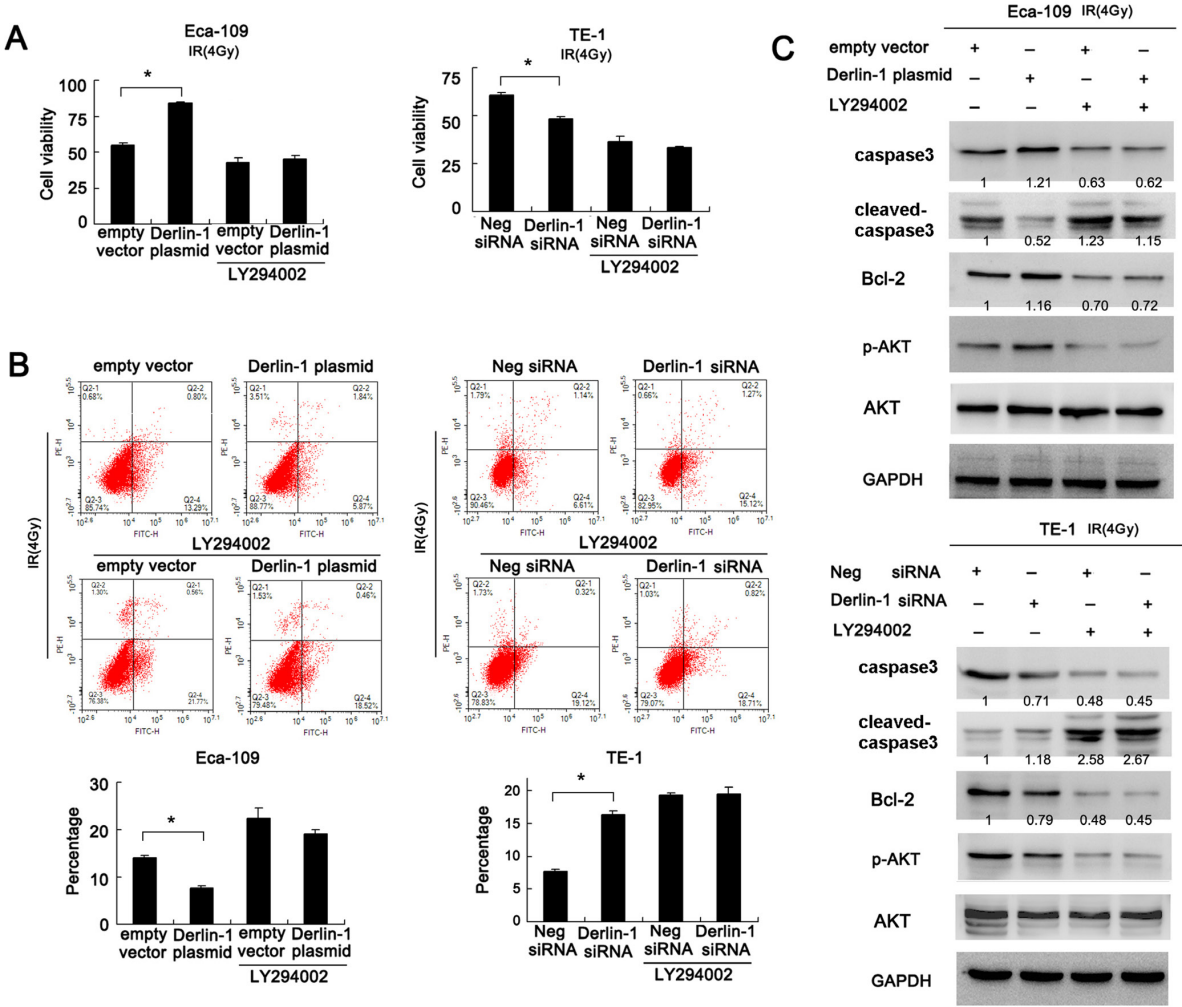
Inhibition of AKT pathway abolishes the role of Derlin-1 on IR induced apoptosis (**A**) CCK-8 assay demonstrated that LY294002 dramatically reduced the effect of Derlin-1 on cell viability. In Eca-109 cells treated with LY294002, Derlin-1 plasmid failed to upregulate cell viability. In TE-1 cell line with LY294002, Derlin-1 siRNA did not cause significant downregulation of cell viability. (**B**) Annexin V/PI staining showed that LY294002 treatment significantly upregulated the percentage of apoptosis. In LY294002 treated Eca-109 cells, the effect of Derlin-1 was not significant as that in untreated group. Similar result was observed in TE-1 cell line with Derlin-1 siRNA. (**C**) AKT inhibition by LY294002 induced caspase3 cleavage and Bcl-2 downregulation. LY294002 also abolished the effect of Derlin-1 and its siRNA on Bcl-2 and cleaved caspase3 in TE-1 and Eca-109 cells. Relative intensity of western blot bands was quantified by ImageJ software. *p < 0.05.

To confirm the involvement of AKT signaling pathway in Derlin-1 induced IR resistance, we adopted AKT inhibitor LY294002. Cells transfected with either Derlin-1 plasmid or siRNA were treated with LY294005 (5 μmol/L). LY294002 effectively blocked AKT phosphorylation in both cell lines. All groups were treated with 4 Gy IR. CCK-8 assay demonstrated that LY294002 dramatically reduced the effect of Derlin-1 on cell viability (Figure 5A). Annexin V/PI staining showed that LY294002 treatment significantly upregulated the percentage of apoptosis. In LY294002 treated Eca-109 cells, the effect of Derlin-1 was not significant as that in untreated group. Similar result was observed in TE-1 cell line with Derlin-1 siRNA (Figure 5B). Next, we examined the effect of LY294002 on caspase3 cleavage and Bcl-2. As shown in Figure 5C, AKT inhibition induced caspase3 cleavage and Bcl-2 downregulation. AKT inhibition also abolished the effect of Derlin-1 and its siRNA on Bcl-2 and cleaved caspase3 in TE-1 and Eca-109 cells. Collectively, these results suggest that Derlin-1 may induce resistance to radiation induced apoptosis by activating AKT/Bcl-2 signaling cascade.

## DISCUSSION

To date, many reports have shown that Derlin-1 overexpression is associated with aggressive phenotype in various cancers including breast, pancreatic and lung cancer [13, 14, 17]. However, its involvement in human ESCC remains unexplored. To explore the role of Derlin-1 in this malignant tumor, first we checked its protein expression in 125 ESCC samples with follow-up data. Our data clearly demonstrated that Derlin-1 levels were elevated in ESCC tissues compared with normal esophageal tissues, which positively correlated with advanced TNM stage and T stage. Survival analysis revealed that Derlin-1 overexpression correlated with resistance to chemoradiotherapy or radiotherapy. In addition, Derlin-1 status serves as an independent predictor for poor patient survival. These data is in accordance with previous studies and indicates a potential role of Derlin-1 in the therapeutic response of esophageal squamous cell carcinoma.

In clinical practice, radiotherapy is widely used in advanced ESCC. Here, 73 patients received radiotherapy and 52 patients received chemoradiotherapy. We divided the population into two groups and analyzed clinical significance of Derlin-1. The results showed that Derlin-1 could serve as prognostic predictor in both patients who receive radiotherapy, with or without chemotherapy. Using plasmid and siRNA transfection, we observed that Derlin-1 overexpression decreased radiosensitivity while Derlin-1 depletion increased sensitivity to IR. This effect was more obvious in cells treated with higher IR dosage, suggesting IR treatment expanded the role of Derlin-1 on cancer cell survival.

Among the mechanisms of IR induced cell death, mitotic catastrophe and apoptosis are the most important ones. Mitotic catastrophe is delayed mitotic-linked cell death which results from premature or inappropriate entry of cells into mitosis which is caused by chemical stimulation or ionizing radiation [19–21]. Here we examined mitotic catastrophe and apoptosis using DAPI staining (to examine micronuclei formation) and Annexin V/PI staining respectively. We found that Derlin-1 overexpression attenuated mitotic catastrophe and apoptosis induced by IR treatment, with downregulation of caspase3 cleavage. Derlin-1 depletion exhibited the opposite effects. Thus, Derlin-1 overexpression could enable cancer cells to overcome apoptosis and mitotic catastrophe, which in turn facilitates therapeutic resistance.

Resistance to radiotherapy involves multiple proteins, some researchers attributed radiation effectiveness to apoptosis related proteins such as p53 and Bcl-2 [22–24]. We examined a panel of relevant targets and found that Bcl-2, an potent apoptosis inhibitor could be upregulated by Derlin-1 overexpression. Bcl-2 is involved in the development of ESCC resistance to IR [25–28]. Based on these results, we believe Derlin-1 suppresses IR induced apoptosis through upregulation of Bcl-2 in ESCC cells.

Then we investigated the potential signaling pathway involved in the regulation of Bcl-2. AKT activation is involved in the induction of both IR resistance and Bcl-2 [29–32]. We showed that Derlin-1 could induce upregulation of AKT phosphorylation. We also performed immunoprecipitation with Derlin-1 antibody, and detected PI3K p110α in the Derlin-1 immunoprecipitates. PI3K functions upstream of AKT and was composed of a catalytic subunit (p110) and a regulatory subunit. PI3K p110 was found to be oncogenic and was implicated in various cancers [33–35]. Moreover, LY294002, an AKT inhibitor, blocked the effects of Derlin-1 on Bcl-2 and IR resistance, indicating that PI3K/AKT/Bcl-2 signaling mediated the biological effects of Derlin-1. Activation of AKT pathway play a major role in the inhibition of apoptosis. Inhibition of the PI3K/AKT pathway could also induce mitotic catastrophe [36]. Thus the biological role of Derlin-1 is dependent on its association with PI3K, which subsequently activate AKT pathway. We also found that Derlin-1 could slightly upregulate ERK phosphorylation. However, its effect on ERK in ESCC cells was not so significant as that in lung cancer cell lines.

In summary, for the first time we describe the clinical significance of Derlin-1 in human ESCC. High Derlin-1 status might be a predictor of cancer aggressiveness and serve as an independent prognostic factor for ESCC patients with chemoradiotherapy. Derlin-1 interacts with p110α and activates AKT/Bcl-2 pathway, which accounts for decreases in radiation induced apoptosis in ESSC cells. Derlin-1 might serve as a novel predictor for radiosensitivity and promising therapeutic target for ESCC.

## MATERIALS AND METHODS

### Patients and specimens

A total of 125 paraffin embedded ESCC specimens were obtained from patients diagnosed with ESCC who underwent pre-therapeutic endoscopy examination at First Affiliated Hospital of China Medical University between January 2006 and January 2014. All patients had received concomitant radiotherapy (A total dose of 60–70 Gy). 52 of the 125 patients received cisplatin based chemotherapy. The histological diagnosis was evaluated according to the WHO 2009 guidelines. Tumor staging was performed according to the TNM classification (6th edition) of the International Union Against Cancer (UICC, 2002). Clinical data was obtained from medical records. 20 fresh ESCC specimens with corresponding normal adjacent tissues were collected for mRNA analysis. The study protocol was approved by the Institutional Review Board of China Medical University.

### Immunohistochemical staining

The paraffin embedded tissue sections were treated with xylene, graded alcohol (100%–75%) and distill water. Then antigen retrieval was carried out in 0.01 M citrate buffer. Tissue sections were treated with hydrogen peroxide and goat serum for 20 minutes. Derlin-1 antibody (1:300, Sigma, USA) was incubated with each section at 4°C overnight. EliVision plus kit from Maixin (Maixin, China) and DAB kit (MaiXin, China) were used for immunohistochemical staining. Counterstaining was performed with hematoxylin. Sections were dehydrated in graded alcohol before mounting. Derlin-1 staining intensity was examined by two pathologists. We evaluated Derlin-1 staining according to a previous report [17]. Briefly, the cytoplasmic intensity of Derlin-1 staining was scored as 0 (no signal), 1 (moderate), 2 (strong). Percentage scores were assigned as 1- 1–25%, 2- 26–50%, 3- 51–75% and 4- 76–100%. The scores of each tumor sample were multiplied to give a final score of 0 to 8, and the tumors were finally determined as having Derlin-1 high expression (overexpression) when the tumor sample reached a score ≥ 4; tumor samples with a score < 4 were considered as having low expression.

### Cell culture and transfection

TE-1, TE-13 and Eca-109 cell lines were obtained from American Type Culture Collection (Manassas, VA, USA). Cells were cultured in DMEM (Invitrogen, Carlsbad, CA, USA) containing 10% FBS (Gibco, Invitrogen, USA). We found that expression of Derlin-1 is high in TE-1 and low in ECa-109 cell line. Thus, we selected these two lines for siRNA and plasmid transfection.

Derlin-1 and empty plasmid was previous described [17]. Lipofectamine 3000 transfection reagent was used for plasmid transfection (Invitrogen, USA). The empty vector (pcDNA5) was used as negative control. Derlin-1 SMARTpool siRNA and Negative control siRNA were obtained from Dharmacon (GE healthcare, USA). DharmaFECT 1 (GE healthcare, USA) was used for siRNA transfection.

### Irradiation treatment

ESCC cells were treated with X-ray irradiation using a linear accelerator (Primus, Siemens, Germany) with a dose of 0, 2, 4, 6 Gy. X-ray irradiation was performed when cell density reached 80%. After irradiation, the cells were harvested and used for further analysis.

### Quantitative real-time PCR

Real-time PCR was performed using SYBR Green master mix (TAKARA, Dalian, China). PCR was performed using 7500 Real-Time PCR System (Applied Biosystems). β-actin was used as the reference gene. The relative expression of target genes were calculated using the 2^-ΔΔCt^ method. The primer sequences are as follow: Derlin-1 forward, 5′CGACTTGAAACAGGAGCTTTTGA 3′, Derlin-1 reverse, 5′ AATCATCAGCAACTGCATATCCAT 3′; β-actin forward, 5′ ATAGCACAGCCTGGATAGCAAC GTAC 3′, β-actin reverse, 5′ CACCTTCTACAATGAGCTG CGTGTG 3′.

### Western blot analysis

Total proteins from cells were extracted in cell lysis buffer and quantified using the Bradford method. Protein samples were transferred to PVDF membranes (Millipore, USA) and incubated overnight at 4°C with primary antibody against Derlin-1 (1: 1000; Sigma), Bcl-2, p-AKT, AKT, caspase3, cleaved caspase3, PI3K p110α, p-ERK, p-GSK3β (1:1000, Cell Signaling Technology, Boston, USA), p-PI3K p85(Y607) (1:600, Abcam, Cambridge, UK) and GAPDH (1:2000; Santa Cruz, USA). After incubation with peroxidase-coupled secondary antibody (1:2000, Santa Cruz, USA) at 37°C for two hours. Target proteins on PVDF membrane were visualized using ECL kit (ThermoFisher, USA) and obtained using DNR Imaging System (DNR, Israel).

### Immunoprecipitation

Magnetic Beads (Bio-Rad) were incubated with antibodies (Derlin-1 and p110α) and unbound antibodies were washed away according to the manufacturer's protocol. Beads-antibody complex was incubated with target protein. The beads were magnetized using SureBeads magnetic rack (Bio-Rad) and supernatant was discarded. Then elution buffer was used to collect purified target protein for western blot analysis.

### CCK-8 cell viability assays

CCK8 assay was performed using Cell Counting Kit-8 solution (Dojindo, Gaithersburg, MD) according to the manufacturer's protocol. Cells were incubated with 10 μl/well of Cell Counting Kit-8 solution during the last 4 hours of the culture. The cell culture plate was measured at 490 nm spectrophotometrically. Experiment was repeated in triplicate.

### Flow cytometry of apoptosis detection

Cell apoptosis detection was performed with Annexin V/PI double staining Kit (BD bioscience, USA). Briefly, 48 hours after transfection, cells were harvested by 0.25% trypsin, washed twice with chill PBS, followed by being resuspended in 250 μl of binding buffer. Staining solution containing Annexin V/FITC and propidium iodide was added in cell suspension. After incubation in the dark for 30 min, the cells were analyzed by BD FACS Calibur flow cytometer (Becton-Dickinson, USA). Experiment was repeated in triplicate.

### Statistical analysis

SPSS 12 for Windows was used for all statistical analyses. A χ^2^ test was used to examine possible correlations between Derlin-1 expression and clinicopathologic factors. Student's *t*-test was used to compare other date. *p* < 0.05 was considered to indicate statistical significance.
